# What would you choose: Online or Offline or Mixed services? Feasibility of online HIV counselling and testing among Thai men who have sex with men and transgender women and factors associated with service uptake

**DOI:** 10.1002/jia2.25118

**Published:** 2018-07-22

**Authors:** Nittaya Phanuphak, Tarandeep Anand, Jureeporn Jantarapakde, Chattiya Nitpolprasert, Kanittha Himmad, Thanthip Sungsing, Deondara Trachunthong, Sangusa Phomthong, Petchfa Phoseeta, Sumitr Tongmuang, Pravit Mingkwanrungruang, Dusita Meekrua, Supachai Sukthongsa, Somporn Hongwiangchan, Nutchanin Upanun, Jiranuwat Barisri, Tippawan Pankam, Praphan Phanuphak

**Affiliations:** ^1^ PREVENTION The Thai Red Cross AIDS Research Centre Bangkok Thailand; ^2^ Service Workers IN Group (SWING) Foundation Bangkok Thailand; ^3^ SWING Foundation Pattaya Chonburi Thailand; ^4^ Rainbow Sky Association of Thailand Bangkok Thailand; ^5^ Sisters Foundation Pattaya Chonburi Thailand; ^6^ Anonymous Clinic Laboratory The Thai Red Cross AIDS Research Centre Bangkok Thailand

**Keywords:** Online, HIV testing, counselling, men who have sex with men, transgender women

## Abstract

**Introduction:**

HIV testing coverage remains low among men who have sex with men (MSM) and transgender women (TGW). We studied characteristics of Thai MSM and TGW who chose online and/or offline platforms for HIV counselling and testing and the feasibility of integrating online technologies and HIV self‐testing to create service options.

**Methods:**

From December 2015 to June 2017, MSM and TGW enrolled from Bangkok Metropolitan Region and Pattaya could choose between: 1 offline HIV counselling and testing (Offline group), 2 online pre‐test counselling and offline HIV testing (Mixed group), and 3 online counselling and online, supervised, HIV self‐testing (Online group). Sociodemographic data, risk behaviour and social network use characteristics were collected by self‐administered questionnaires. Logistic regression models identified covariates for service preferences.

**Results:**

Of 472 MSM and 99 TGW enrolled, 202 self‐selected the Offline group, 158 preferred the Mixed group, and 211 chose the Online group. The Online group had the highest proportion of first‐time testers (47.3% *vs*. 42.4% *vs*. 18.1%, *p* < 0.001) and reported highest HIV prevalence (15.9% *vs*. 13.0% *vs*. 3.4%, *p* = 0.001) as compared to Offline and Mixed groups, respectively. Having tested for HIV twice or more (OR 2.57, 95% CI 1.03 to 6.41, *p* = 0.04) increased the likelihood to choose online pre‐test counselling. Being TGW (OR 6.66, 95% CI 2.91 to 15.25, *p* < 0.001) and using social media from four to eight hours (OR 2.82, 95% CI 1.48 to 5.37, *p* = 0.002) or >8 hours (OR 2.33, 95% CI 1.05 to 5.16, *p* = 0.04) increased selection of online, supervised, HIV self‐testing. Providers primarily used smartphones (79.2%) and laptops (37.5%) to deliver online services. Self‐testing strip image sharpness and colour quality were rated “good” to “excellent” by all providers. Most participants (95.1%) agreed that online supervision and HIV self‐testing guidance offered were satisfactory and well delivered.

**Conclusions:**

Online HIV services among MSM and TGW are feasible in Thailand and have the potential to engage high proportions of first‐time testers and those with high HIV prevalence. When designing public health interventions, integrating varied levels of online HIV services are vital to engage specific sections of MSM and TGW populations in HIV services.

**Clinical Trial Number:**

NCT03203265

## 1 Introduction

HIV testing is the first critical entry point into HIV prevention and treatment cascades. However, scaling‐up HIV testing among key populations (KPs), including men who have sex with men (MSM), transgender women (TGW), sex workers and people who inject drugs, remains a global challenge 1. In Thailand, MSM and TGW contribute to more than half of new HIV cases annually 2. Recent estimate demonstrated that only 29% of MSM had received an HIV test in the past 12 months 3. Although country data for TGW is limited, HIV testing coverage was reported at only 34% in 2014 3.

A survey conducted among 4639 Thai MSM and TGW during 2010 to 2011 identified fear of testing (60%), not recognizing risk exposure (40%), and unavailability of friendly testing services (15%) as main reasons for never testing for HIV 4. In response, Thailand with support from the USAID has implemented the Key Population‐Led Health Services (KPLHS) model led by community‐based organizations (CBOs) serving MSM and/or TGW. KPLHS are a defined set of HIV‐related health services delivered by trained KP community health workers in partnership with other health sector entities. This KPLHS model has proved extremely successful in engaging MSM and TGW who are at high‐risk for HIV infection into early diagnosis, early antiretroviral treatment (ART) linkage, and high pre‐exposure prophylaxis (PrEP) uptake 5. In 2017 alone, KP community health workers contributed to 38% of the 41,386 HIV counseling and testing services and 26% of 4840 new HIV diagnoses among MSM and TGW in Thailand 6.

Given the heterogeneity in psychosocial context and health and digital literacy among MSM and TGW, multiple HIV testing options are needed to ensure that certain sub‐populations are not excluded 7. Motivation and education‐based interventions through peer mobilizers and mass media campaigns could help tackle fear of testing. New technologies including online communication platforms and HIV self‐testing have shown potential to overcome structural barriers and increase access to HIV counselling and testing among KPs 8, 9.

A systematic review revealed that supervised HIV self‐testing (conducted with real‐time support from a healthcare provider) and unsupervised HIV self‐testing were highly acceptable and preferred among people at risk for HIV infection, although lower sensitivity was found when self‐testing was unsupervised 10 Online support to perform HIV self‐testing has shown advantage over unsupervised HIV self‐testing by the ability to address certain concerns such as lack of pre‐ and post‐test counseling 11. Thai MSM and TGW who lead the region in Internet and technology adoption and utilization have shown consistently high preferences for online HIV service delivery 12, 13. Online supervised self‐testing with special emphasis on individual‐level counselling could help address barriers to self‐testing uptake among Thai MSM, such as fear of receiving self‐testing kit at home, fear of one's own lack of understanding of self‐testing and receiving results alone 14.

We leveraged Online, Offline and Mixed HIV counselling and testing, three distinct service delivery models by integrating online and HIV self‐testing technologies, KPLHS and public healthcare services. In this paper, we specifically explored characteristics of Thai MSM and TGW as well as key factors they took into account when choosing service options. In addition, we studied providers’ technology skills and utilization levels and their feasibility of delivering online HIV services as well as satisfaction among MSM and TGW clients receiving services.

## 2 Methods

From December 2015 to June 2017, Thai MSM and TGW were consecutively recruited and enrolled into a 12‐month cohort study, with six‐monthly visits to assess the preferences and feasibility of online and offline HIV counselling and testing strategies (NCT03203265). The Institutional Review Board of the Faculty of Medicine, Chulalongkorn University, and the Bangkok Metropolitan Administration Ethics Committee approved this study. Inclusion criteria included Thai national, aged >18 years, being men or TGW, engaged in unprotected anal sex with men at least once in the past 6 months, living in Bangkok Metropolitan Region or Pattaya, and not known to be HIV positive. The study was supported by amfAR GMT Initiative grant and conducted by the Thai Red Cross AIDS Research Centre (TRCARC), Service Workers IN Group (SWING) Foundation, Rainbow Sky Association of Thailand (RSAT), and Sisters Foundation. SWING, RSAT and Sisters were CBOs serving MSM and TGW. SWING had two community health centres in Bangkok and Pattaya, RSAT had one community health centre in Bangkok, and Sisters had one in Pattaya. We reported baseline data from this study.

### 2.1 Recruitment strategies and informed consent process

Online recruitment strategies included dissemination of study recruitment posters, text‐based messages, and an online HIV self‐testing video promoted through organizations’ websites, banners on popular websites, and platforms commonly used by MSM and TGW such as Facebook, LINE chat groups, Camfrog video chat rooms, Hornet, and Jack'D. Offline recruitment was conducted by CBOs at hot spots using study posters and flyers. Participants interested in joining the study were scheduled for either online or offline informed consent process, based on individual preference. The online informed consent process was conducted using a real‐time video chatting platform enabling the participant information sheet to be reviewed via shared screen.

### 2.2 HIV counselling and testing via online and/or offline strategies

**Figure 1:**
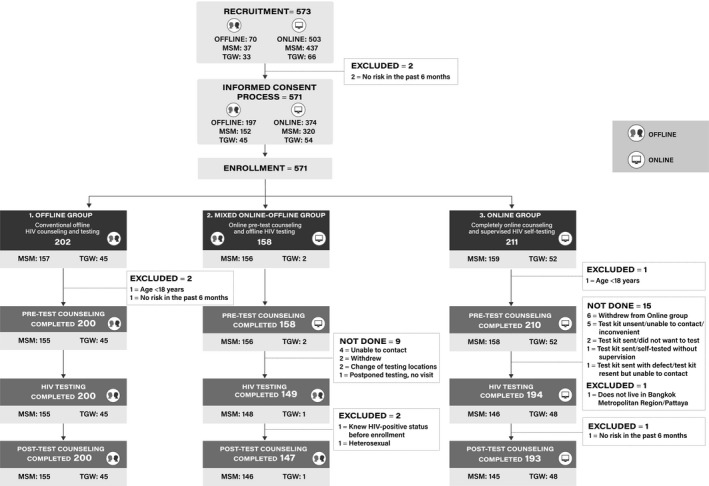
Flow of recruitment and enrolment of men who have sex with men (MSM) and transgender women (TGW) participants.

To understand the types of service delivery models that appeal to various sub‐groups of MSM and TGW participants, the study allowed participants to self‐select from three distinct strategies (Figure 1) including: (1) conventional offline HIV counselling and testing (Offline group), (2) online pre‐test counselling and offline HIV testing (Mixed group), and (3) completely online counselling and supervised HIV self‐testing (Online group). Services in the Offline group were delivered by staff at TRCARC and the four community health centres. In the Mixed group, pre‐test counselling was conducted online by study staff and participants were scheduled to receive HIV testing and post‐test counselling at a by‐appointment‐only clinic in Bangkok operated by TRCARC. Online group participants received totally online pre‐test counselling, HIV testing, and post‐test counselling. Study staff provided linkage to care and ART initiation support to all HIV‐positive participants, regardless of CD4 count as per the Thailand National Guidelines 15.

### 2.3 HIV testing and other services provided in Online, Mixed and Offline groups

Blood samples were collected by venipuncture in the Offline group for HIV testing. Other sexually transmitted infection testing were conducted based on provider's judgment. HIV testing was conducted according to Thailand National Guidelines 15, starting with either machine‐based 4th generation (at TRCARC) or rapid third generation assay (at the community health centers) with confirmation of reactive result made by another two assays. Confirmed HIV status was available within one to two hours. To identify acute HIV infection cases, all non‐reactive samples were also sent for nucleic acid testing (NAT) by Aptima HIV‐1 RNA qualitative assay (Gen‐Probe Inc., San Diego, CA, USA).

Participants in the Mixed group were scheduled to receive fingerprick blood collection at the clinic. Rapid third generation assay was performed with confirmation of reactive result by another two assays which confirmed HIV status within one to two hours.

In the Online group, participants had an HIV self‐testing package and an online HIV self‐testing video URL link 16 sent to them on a pre‐scheduled date. Study staff contacted the participants to ensure package delivery and scheduled date and time for the online, supervised, HIV self‐testing process. Step‐by‐step, real‐time guidance was provided through a video chatting platform preferred by the participants. For self‐testing, we used a rapid third generation assay (Alere Determine™ HIV 1/2, Alere Medical Co., Ltd., Matsuhidal, Matsudo‐shi, Chiba, Japan) and fingerprick blood sample which allowed for 15 minutes diagnosis result. Post‐test counselling for participants with reactive results emphasized immediate linkage to confirmation and ART initiation at preferred hospitals or clinics.

PrEP and post‐exposure prophylaxis (PEP) were offered to all participants and assistance to access PrEP and PEP services offline was given by study staff. Participants in the Mixed and the Online groups were also provided access to the Adam's Love Electronic health record (EHR) system which enabled secure access to laboratory results, post‐test summaries and appointment scheduling for HIV test, as previously described 17.

### 2.4 Data collection and questionnaires

A self‐administered questionnaire was used at baseline visit to collect sociodemographic, risk behaviour, social network use characteristics, perceived barriers and facilitators for HIV testing, and experiences around stigma and discrimination. Online group participants also completed a questionnaire assessing their decision‐making and reasons for choosing online, supervised, HIV self‐testing, over clinic‐based testing, and feelings post‐service utilization. Study staff delivering online services completed a questionnaire that assessed their technology skills and utilization, and the feasibility and acceptability of delivering online HIV services.

### 2.5 Statistical analysis

Data from the questionnaires were reported overall and by self‐selected groups and by gender as frequency and proportion for categorical parameters and mean (standard deviation, SD) and median (interquartile range, IQR) for continuous parameters. Comparison of continuous variables between groups was made by Two‐sample *t* test, ANOVA techniques or non‐parametric tests. Chi‐square or Fisher's exact test was used for comparison of proportion of characteristics between groups.

The associations of covariates with a preference for online *vs*. offline pre‐test counselling service and online *vs*. offline HIV testing and post‐test counselling services were modelled using binary logistic regression, adjusting for confounders as appropriate. Factors showing significant level of 0.10 in the univariate model were adjusted in the multivariate model.

## 3 Results

The study recruited 437 MSM and 66 TGW through online and 37 MSM and 33 TGW via offline channels (Figure 1). Informed consent process was conducted online with 320 MSM and 54 TGW and offline with 152 MSM and 45 TGW. Of 571 participants enrolled, 202 selected the Offline group, 158 chose the Mixed group, and 211 preferred Online group. Seven individuals were further excluded (Figure 1) and data analyses were subsequently performed on 564 participants (Table 1). Mean (SD) age was 27.9 (7.2) years. In the past six months, 68.3% had multiple sex partners, 77.4% inconsistently used condoms, 6.2% used amphetamine‐type stimulants, and 18.2% engaged in group sex. Basic characteristics of MSM and TGW participants are summarized in Table 1.

**Table 1:** Characteristics of men who have sex with men (MSM) and transgender women (TGW) participants

Characteristics	Overall (N = 564)	MSM (n = 465)	TGW (n = 99)	*p*‐value
1. Demographic data				
Age (years)				
Mean (SD)	27.9 (7.2)	28.1 (7.2)	26.8 (7.2)	0.11^a^
Age 18 to 25	235 (41.7)	192 (41.3)	43 (43.4)	0.69^c^
Age >25	329 (58.3)	273 (58.7)	56 (56.6)	
Education				<0.001^c^
Less than Bachelor degree	221 (47.3)	163 (42.7)	58 (68.2)	
Bachelor degree/above	246 (52.7)	219 (57.3)	27 (31.8)	
Main occupation				<0.001^c^
Unemployed/Student	121 (26)	102 (26.8)	19 (22.6)	
Employed	294 (63.2)	256 (67.2)	38 (45.2)	
Service worker	50 (10.8)	23 (6)	27 (32.1)	
Income (USD)				<0.001^d^
<429	171 (37.6)	127 (34.2)	44 (52.4)	
429 to 857	192 (42.2)	156 (42.1)	36 (42.9)	
858 to 1429	59 (13)	57 (15.4)	2 (2.4)	
1430 to 2857	28 (6.2)	27 (7.3)	1 (1.2)	
≥2858	5 (1.1)	4 (1.1)	1 (1.2)	
2. Using social media				
Do you always use social media?				>0.99^d^
No	23 (5)	19 (5)	4 (4.8)	
Yes	437 (95)	358 (95)	79 (95.2)	
Which social media do you always use? (can select more than one choice)				
Facebook	431 (91.7)	351 (91.2)	80 (94.1)	0.37^c^
Line	427 (90.9)	355 (92.2)	72 (84.7)	0.03^c^
WhatsApp	55 (11.7)	38 (9.9)	17 (20)	0.009^c^
Instagram	225 (47.9)	186 (48.3)	39 (45.9)	0.69^c^
YouTube	325 (69.2)	272 (70.7)	53 (62.4)	0.13^c^
Twitter	172 (36.6)	148 (38.4)	24 (28.2)	0.08^c^
Skype/FaceTime	52 (11.1)	38 (9.9)	14 (16.5)	0.08^c^
Google	101 (21.5)	87 (22.6)	14 (16.5)	0.21^c^
How long do you spend time on social media daily? (excluding playing game)				
Weekday				0.02^c^
less than 2 hours	36 (7.8)	27 (7.1)	9 (10.8)	
2 to 4 hours	141 (30.5)	126 (33.2)	15 (18.1)	
4 to 8 hours	195 (42.1)	150 (39.5)	45 (54.2)	
8 to 24 hours	91 (19.7)	77 (20.3)	14 (16.9)	
Weekend				0.18^c^
less than 2 hours	25 (5.5)	18 (4.8)	7 (9)	
2 to 4 hours	111 (24.5)	98 (26.1)	13 (16.7)	
4 to 8 hours	212 (46.7)	175 (46.5)	37 (47.4)	
8 to 24 hours	106 (23.4)	85 (22.6)	21 (26.9)	
How do you rate your social media skills?				0.08^d^
Excellent	111 (24)	97 (25.5)	14 (16.9)	
Good	198 (42.8)	166 (43.7)	32 (38.6)	
Intermediate	144 (31.1)	110 (29)	34 (41)	
Poor	8 (1.7)	5 (1.3)	3 (3.6)	
No ability	2 (0.4)	2 (0.5)	0 (0)	
Have you ever sought sexual partner on social media?				0.08^c^
No	52 (11.1)	38 (10)	14 (16.5)	
Yes	415 (88.9)	344 (90.1)	71 (83.5)	
If yes, which social media have you used for seeking sexual partner? n = 415				
Facebook	236 (50.2)	183 (47.5)	53 (62.4)	0.01^c^
Applications, e.g., Grindr, Jack'D, Hornet	285 (60.6)	266 (69.1)	19 (22.4)	<0.001^c^
Camfrog	92 (19.6)	75 (19.5)	17 (20)	0.91^c^
Instagram	41 (8.7)	35 (9.1)	6 (7.1)	0.55^c^
Others	10 (2.1)	5 (1.3)	5 (5.9)	–
What device(s) do you use for social media?				
Mobile phone	417 (88.7)	346 (89.9)	71 (83.5)	0.09^c^
Tablet	86 (18.3)	81 (21)	5 (5.9)	0.001^c^
Personal computer (PC)	109 (23.2)	97 (25.2)	12 (14.1)	0.03^c^
Notebook/laptop	148 (31.5)	136 (35.3)	12 (14.1)	<0.001^c^
3. Risk behaviour at baseline				
Age at first sex (years)				
Median (IQR)	17 (15 to 19)	18 (16 to 20)	15 (14 to 18)	<0.001^b^
Perceived HIV risk in the past 6 months				0.44^c^
No risk	50 (10.4)	39 (9.8)	11 (13.3)	
Mild	183 (37.9)	157 (39.3)	26 (31.3)	
Moderate	179 (37.1)	148 (37)	31 (37.4)	
High	71 (14.7)	56 (14)	15 (18.1)	
Median (IQR) number of sexual partners in the past 6 months	4 (2 to 6)	3 (2 to 6)	5.5 (2 to 15)	<0.001^b^
Condom use in the past 6 months				0.61^c^
Never	46 (9.8)	37 (9.5)	9 (11.1)	
Sometime	317 (67.6)	260 (67)	57 (70.4)	
Always	106 (22.6)	91 (23.5)	15 (18.5)	
Drug use in the past 6 months				>0.99^c^
No	304 (64.7)	249 (64.7)	55 (64.7)	
Yes	166 (35.3)	136 (35.3)	30 (35.3)	
Amphetamine‐type stimulants used	29 (6.2)	25 (6.5)	4 (4.7)	0.54^c^
Had group sex in the past 6 months				0.057^c^
No	396 (81.8)	322 (80.3)	74 (89.2)	
Yes	88 (18.2)	79 (19.7)	9 (10.8)	
Yes, median (IQR) times of group sex	2 (1 to 3)	2 (1 to 3)	2 (1 to 3)	–
Yes, median (IQR) number of partners during each group sex	3 (3 to 4)	3 (3 to 4)	3 (3 to 3.5)	–
4. Perceived barriers and facilitators for HIV testing				
Have you ever been tested for HIV before participating in the study?				0.30^c^
No	172 (36.8)	137 (35.7)	35 (41.7)	
Yes	296 (63.3)	247 (64.3)	49 (58.3)	
If yes, median (IQR) times of HIV testing, n = 296	2 (1 to 4)	2 (1 to 4)	1 (1 to 2)	<0.001^b^
Does Nucleic Acid Testing (NAT) influence your decision to test for HIV this time?				0.67^c^
No	139 (30.1)	115 (30.4)	24 (28.6)	
Yes	177 (38.3)	147 (38.9)	30 (35.7)	
Not sure	146 (31.6)	116 (30.7)	30 (35.7)	
What is/are the barrier(s) to HIV testing?				
Inconvenience in travelling to get the service	149 (31.8)	131 (34.1)	18 (21.4)	0.02^c^
Unattractive/not beautiful place	23 (4.9)	17 (4.4)	6 (7.1)	0.30^c^
Inconvenient service hours	140 (29.9)	119 (31)	21 (25)	0.28^c^
Unfriendly staff	51 (10.9)	47 (12.2)	4 (4.8)	0.046^c^
Concern about confidentiality of HIV result	112 (23.9)	96 (25)	16 (19.1)	0.25^c^
Afraid of getting HIV‐positive result	144 (30.8)	113 (29.4)	31 (36.9)	0.18^c^
Never think about HIV testing before	67 (14.3)	62 (16.2)	5 (6)	0.02^c^
Afraid of meeting people you may know	97 (20.7)	89 (23.2)	8 (9.5)	0.005^c^
What is/are facilitator(s) for HIV testing?				
Quality standard of HIV testing service	266 (56.8)	238 (62)	28 (33.3)	<0.001^c^
Clinic hygiene	208 (44.4)	184 (47.9)	24 (28.6)	0.001^c^
Friendly staff	296 (63.3)	254 (66.2)	42 (50)	0.005^c^
Free HIV testing	303 (64.7)	266 (69.3)	37 (44.1)	<0.001^c^
Souvenir after HIV testing	134 (28.6)	122 (31.8)	12 (14.3)	0.001^c^
Online HIV testing	177 (37.8)	153 (39.8)	24 (28.6)	0.054^c^
HIV acquisition knowledge score (9 points)				
Mean (SD)	7.9 (1.3)	8.0 (1.2)	7.6 (1.6)	
Median (IQR)	8 (7 to 9)	8 (7 to 9)	8 (6 to 9)	0.12^b^
HIV prevention knowledge score (8 points)				
Mean (SD)	5.1 (1.5)	5.2 (1.4)	4.9 (1.8)	
Median (IQR)	5 (4 to 6)	5 (4 to 6)	5 (3 to 7)	0.13^b^
Have you ever known someone close to you who is HIV‐positive?				0.62^c^
No	234 (53.6)	200 (54.1)	34 (50.8)	
Yes	148 (33.9)	122 (33)	26 (38.8)	
Not sure/do not know	55 (12.6)	48 (13)	7 (10.5)	
What is/are your attitude(s) about HIV testing?				
I am afraid of needles.	64 (13.7)	55 (14.3)	9 (10.7)	0.38^c^
I am concerned about confidentiality.	169 (36.1)	143 (37.2)	26 (31)	0.28^c^
I think there is less benefit than harm of knowing HIV status.	69 (14.7)	56 (14.6)	13 (15.5)	0.83^c^
I want a test which will detect HIV soonest after the exposure.	255 (54.5)	224 (58.3)	31 (36.9)	<0.001^c^
I want home testing.	152 (32.5)	130 (33.9)	22 (26.2)	0.17^c^
I think HIV testing is a good way to take care of one's health.	265 (56.6)	223 (58.1)	42 (50)	0.18^c^
Do you want to confirm HIV status and/or link to ART if tested reactive/positive? (n = 426)				0.08^d^
Yes, immediately	362 (86.8)	310 (87.6)	52 (82.5)	
Within 1 month	41 (9.8)	34 (9.6)	7 (11.1)	
1 to 3 months	8 (1.9)	5 (1.4)	3 (4.8)	
3 to 6 months	5 (1.2)	5 (1.4)	0 (0)	
5. Stigma and discrimination related to HIV				
Family disclosure of gender identity				<0.001^c^
Yes, self disclosure	228 (47.6)	178 (44.8)	50 (61)	
Yes, non‐self disclosure	128 (26.7)	97 (24.4)	31 (37.8)	
No	123 (25.7)	122 (30.7)	1 (1.2)	
Discrimination within family due to gender identity				<0.001^c^
No	292 (70.4)	235 (70.6)	57 (69.5)	
Yes, current	9 (2.2)	7 (2.1)	2 (2.4)	
Yes, past	45 (10.8)	27 (8.1)	18 (22)	
Do not know/not sure	69 (16.6)	64 (19.2)	5 (6.1)	
In the past 12 months, ever been rejected from workplace due to gender identity				<0.001^c^
No	434 (91)	378 (95.2)	56 (70)	
Yes	43 (9)	19 (4.8)	24 (30)	
Feel embarrassed due to gender identity				0.01^d^
Yes, definitely	9 (1.9)	9 (2.3)	0 (0)	
Yes, maybe	26 (5.5)	23 (5.8)	3 (3.6)	
Probably not	96 (20.1)	88 (22.3)	8 (9.6)	
Definitely not	346 (72.5)	274 (69.5)	72 (86.8)	
In the past 12 months, ever been sexually abused				<0.001^c^
No	328 (68.9)	285 (72.5)	43 (51.8)	
Yes	148 (31.1)	108 (27.5)	40 (48.2)	
In the past 12 months, ever been physically abused				<0.001^d^
No	456 (95.2)	386 (97.2)	70 (85.4)	
Yes	23 (4.8)	11 (2.8)	12 (14.6)	
In the past 12 months, ever experienced stigma and discrimination in healthcare setting (n = 169)				
Denied services	5 (3)	5 (3.5)	0 (0)	–
Sub‐standard services	13 (7.8)	10 (6.9)	3 (13)	0.39^d^

a *p*‐value for comparison of mean of characteristic between group (Two‐sample *t* test).

b *p*‐value for comparison of median of characteristics between group (Mann–Whitney two‐statistic).

c *p*‐value for comparison of proportion of characteristics between group (Chi‐square test).

d *p*‐value for comparison of proportion of characteristics between group (Fisher's Exact test).

SD, standard deviation; IQR, interquartile range; MSM, men who have sex with men; TGW, transgender women; USD, United States dollar; ART, antiretroviral therapy.

### 3.1 First‐time tester rate and HIV prevalence by self‐selected groups

All participants in the Offline group completed HIV testing process although proportions were lower in the Mixed group (94.3%) and Online group (92.4%) (Figure 1). Nine participants in the Mixed group and 11 in the Online group did not show up for the offline or the online scheduled visit, and four could not complete online, supervised, self‐testing process.

First‐time testing rate was 42.4% in the Offline group, 18.1% in the Mixed group, and 47.3% in the Online group, *p* < 0.001. HIV prevalence was lowest in the Mixed group followed by the Offline and Online groups (3.4% *vs*. 13% *vs*. 15.9%, *p* = 0.001). One HIV‐positive case was diagnosed by NAT in the Offline group. The Mixed group also had lowest proportion of TGW when compared to the Offline and Online groups (1.3% *vs*. 22.5 *vs*. 25.0%, *p* < 0.001).

### 3.2 Factors associated with selecting “online pre‐test counselling” (Table 2)

**Table 2:** Factors associated with the selection of online pre‐test counselling

Factors	Overall (N = 564)			MSM (n = 465)			TGW (n = 99)		
	aOR	95% CI	*p*‐value	aOR	95% CI	*p*‐value	aOR	95% CI	*p*‐value
Demographic									
Marital status									
Single	–	–	–	1	ref		–	–	–
Living together with male partner	–	–	–	2.20	1.06 to 4.57	0.04	–	–	–
Living together with female partner/others	–	–	–	1.03	0.25 to 4.23	0.97	–	–	–
Education									
Less than Bachelor degree	1	ref		1	ref		–	–	–
Bachelor degree/above	2.02	1.02 to 4.00	0.045	2.77	1.33 to 5.77	0.007	–	–	–
Main occupation									
Unemployed/student	1	ref		–	–	–	–	–	–
Employed	0.38	0.16 to 0.89	0.03	–	–	–	–	–	–
Service worker	0.15	0.04 to 0.53	0.003	–	–	–	–	–	–
Income (USD)									
<429	1	ref		–	–	–	–	–	–
429 to 857	2.31	1.02 to 5.21	0.04	–	–	–	–	–	–
≥858	1.81	0.64 to 5.10	0.27	–	–	–	–	–	–
Main social media/search engine platform used									
Facebook	1.02	0.35 to 2.96	0.97	–	–	–	–	–	–
Line	–	–	–	–	–	–	4.50	0.54 to 37.31	0.16
Instagram	2.22	1.12 to 4.38	0.02	3.97	1.90 to 8.29	<0.001	–	–	–
YouTube	1.20	0.59 to 2.46	0.62	–	–	–	3.72	0.69 to 19.93	0.13
Skype/FaceTime	–	–	–	–	–	–	0.16	0.02 to 1.43	0.10
Google	0.41	0.20 to 0.85	0.02	0.34	0.15 to 0.75	0.007	–	–	
Social media platform for seeking online sex partner									
Applications, e.g., Grindr, Jack'D, Hornet	–	–	–	–	–	–	4.98	0.71 to 35.00	0.11
Instagram	0.27	0.10 to 0.74	0.01	0.21	0.06 to 0.71	0.01	–	–	–
Device(s) used for social media									
Notebook/laptop	1.66	0.84 to 3.29	0.15	–	–	–	–	–	–
Age at first sex >17 years	–	–	–	2.43	1.18 to 4.98	0.02	–	–	–
Ever been tested for HIV									
No	1	ref		1	ref		–	–	–
Yes, ≤2 times	0.96	0.47 to 1.97	0.92	0.85	0.38 to 1.90	0.70	–	–	–
Yes, >2 times	2.57	1.03 to 6.41	0.04	3.21	1.10 to 9.37	0.03	–	–	–
NAT influencing decision to get HIV testing									
No/not sure	–	–	–	–	–	–	1	ref	
Yes	–	–	–	–	–	–	0.13	0.02 to 0.76	0.02
Barriers to HIV testing									
Unattractive/not beautiful place	–	–	–	0.06	0.01 to 0.36	0.002	–	–	–
Inconvenient service hours	1.71	0.89 to 3.30	0.11	2.92	1.32 to 6.45	0.008	–	–	–
Afraid of knowing HIV‐positive result	–	–	–	–	–	–	0.13	0.03 to 0.64	0.01
Facilitators for HIV testing									
Quality standard of HIV testing service	0.41	0.20 to 0.86	0.02	0.34	0.14 to 0.81	0.02	–	–	–
Clinic hygiene	0.33	0.16 to 0.66	0.002	0.49	0.22 to 1.10	0.08	0.14	0.01 to 0.28	0.001
Souvenir after HIV testing	1.91	0.93 to 3.90	0.08	2.04	0.88 to 4.74	0.10	–	–	–
Online HIV testing	2.68	1.33 to 5.39	0.006	2.64	1.19 to 5.89	0.02	8.80	1.05 to 73.61	0.045
HIV prevention knowledge score	1.77	0.94 to 3.31	0.08	1.87	0.92 to 3.82	0.09	–	–	–
Know someone close is HIV positive									
No	1	ref		1	ref		**–**	**–**	**–**
Yes	0.43	0.22 to 0.84	0.01	0.29	0.13 to 0.65	0.002	**–**	**–**	**–**
Not sure/do not know	0.61	0.24 to 1.51	0.28	0.54	0.19 to 1.55	0.26	**–**	**–**	**–**
Attitudes towards HIV testing									
Less benefit than harm of knowing HIV status	0.41	0.18 to 0.94	0.04	0.46	0.17 to 1.24	0.12	**–**	**–**	**–**
Want home testing	1.94	0.97 to 3.90	0.06	2.18	0.96 to 4.94	0.06	**–**	**–**	**–**
HIV testing as a good way to take care of one's health	**–**	**–**	**–**	**–**	**–**	**–**	8.75	1.56 to 49.01	0.01
Family disclosure of gender identity									
No	1	ref		1	ref		**–**	–	**–**
Yes, self disclosure	6.60	1.95 to 22.35	0.002	11.28	2.62 to 48.5	0.001	**–**	**–**	**–**
No, non‐self disclosure	18.45	4.98 to 68.31	<0.001	14.21	7.09 to 65.08	<0.001	**–**	**–**	**–**
Discrimination within family									
No	1	ref		1	ref		**–**	**–**	**–**
Yes, current and past	3.45	1.14 to 10.44	0.03	10.37	2.10 to 51.21	0.004	**–**	**–**	**–**
Do not know/not sure	2.44	0.85 to 7.04	0.10	2.72	0.80 to 9.26	0.11	**–**	**–**	**–**
Feel embarrassed due to gender identity									
Definitely not	**–**	**–**	**–**	1	ref		**–**	**–**	**–**
Yes definitely/yes maybe/probably not	**–**	**–**	**–**	1.66	0.73 to 3.77	0.23	**–**	**–**	**–**
In the past 12 months, ever been sexually abused									
No	1	ref		**–**	**–**	**–**	**–**	**–**	**–**
Yes	1.02	0.49 to 2.12	0.96	**–**	**–**	–	**–**	**–**	–
In the past 12 months, ever experienced stigma and discrimination in healthcare setting due to gender identity									
Sub‐standard services	0.16	0.02 to 1.43	0.10	0.06	0.00 to 0.80	0.03	**–**	**–**	**–**

aOR, adjusted odds ratio; CI, confidence interval; TGW, transgender women; MSM, men who have sex with men; USD, United States dollar.

Models were run separately for overall, MSM and TGW. Factors showing significant level of 0.10 in univariate model were adjusted in multivariate models for each group. Factors not included in multivariate models were shown as – in this table.

In a logistic regression model, having a bachelor degree or higher (OR 2.02, 95% CI 1.02 to 4, *p* = 0.045), having monthly income between 429 and 857 USD (OR 2.31, 95% CI 1.02 to 5.21, *p* = 0.04), and having had HIV tested twice or more (OR 2.57, 95% CI 1.03 to 6.41, *p* = 0.04) increased online pre‐test counselling selection chances. Identifying inconvenient service hours as a barrier to HIV testing (OR 2.92, 95% CI 1.32 to 6.45, *p* = 0.008) influenced MSM to choose online pre‐test counselling. Among TGW, positive attitude towards HIV testing (OR 8.75, 95% CI 1.56 to 49.01, *p* = 0.01) was associated with a higher chance to select online pre‐test counselling, although prioritizing NAT‐based HIV testing (OR 0.13, 95% CI 0.02 to 0.76, *p* = 0.02) decreased such chances.

### 3.3 Factors associated with selecting “online HIV testing and online post‐test counseling” (Table 3)

**Table 3:** Factors associated with the selection of online HIV testing and post‐test counselling

Factors	Overall (N = 564)			MSM (n = 465)			TGW (n = 99)		
	aOR	95% CI	*p*‐value	aOR	95% CI	*p*‐value	aOR	95% CI	*p*‐value
Gender									
MSM	1	ref		–	–	–	–	–	–
TGW	6.66	2.91 to 15.25	<0.001	–	–	–	–	–	–
Main social media/search engine platform used									
WhatsApp	–	–	–	0.19	0.05 to 0.72	0.02	–	–	–
YouTube	–	–	–	–	–	–	6.88	1.59 to 29.81	0.01
Skype/face time	0.45	0.17 to 1.2	0.11	–	–	–	–	–	–
Time spent on social media on weekday?									
<4 hours	1	ref		–	–	–	–	–	–
4 to 8 hours	2.82	1.48 to 5.37	0.002	–	–	–	–	–	–
8 to 24 hours	2.33	1.05 to 5.16	0.04	–	–	–	–	–	–
Main device used for social media									
Personal computer (PC)	0.47	0.23 to 0.96	0.04	–	–	–	–	–	–
Age at first sex >17 years	–	–	–	4.39	1.95 to 9.89	<0.001	–	–	–
Perceived HIV risk in the past 6 months									
No risk	–	–	–	1	ref		–	–	–
Mild	–	–	–	0.23	0.08 to 0.7	0.01	–	–	–
Moderate	–	–	–	0.47	0.16 to 1.41	0.18	–	–	–
High	–	–	–	0.34	0.08 to 1.42	0.14	–	–	–
NAT influencing decision to get HIV testing									
No/Not sure	–	–	–	1	ref		1	ref	
Yes	–	–	–	0.42	0.20 to 0.90	0.03	0.11	0.02 to 0.54	0.007
Barriers to HIV testing									
Inconvenient service hours	1.82	0.93 to 3.59	0.08	–	–	–	–	–	–
Concern about confidentiality of HIV result	–	–	–	2.99	1.32 to 6.76	0.009	–	–	–
Afraid of knowing HIV‐positive result	–	–	–	–	–	–	0.16	0.04 to 0.67	0.01
Facilitators for HIV testing									
Quality standard of HIV testing service	0.42	0.22 to 0.81	0.01	0.38	0.17 to 0.84	0.02	0.17	0.02 to 1.56	0.12
Clinic hygiene	0.31	0.16 to 0.60	<0.001	0.42	0.18 to 0.99	0.047	0.10	0.01 to 0.86	0.04
Friendly staff	–	–	–	0.56	0.25 to 1.23	0.15	–	–	–
Free HIV testing	–	–	–	0.47	0.21 to 1.03	0.06	–	–	–
Online HIV testing	5.73	2.99 to 10.98	<0.001	6.30	2.87 to 13.86	<0.001	13.46	1.72 to 105.08	0.01
Attitudes towards HIV testing									
Afraid of needles	–	–	–	1.95	0.79 to 4.79	0.15	–	–	–
Want a test which will detect HIV soonest after the exposure	0.30	0.16 to 0.57	<0.001	0.29	0.13 to 0.62	0.002	–	–	–
Want home testing	6.00	3.10 to 11.63	<0.001	10.64	4.60 to 24.63	<0.001	–	–	–
HIV testing as a good way to take care of one's health	–	–	–	–	–	–	8.73	1.78 to 42.76	0.008
Intention to confirm HIV status and/or start ART after reactive/positive test result									
No/not immediately	1	ref		1	ref		–	–	–
Immediately	0.32	0.15 to 0.67	0.003	0.23	0.09 to 0.59	0.002	–	–	–
In the past 12 months, ever been sexually abused									
No	1	ref		–	–	–	–	–	–
Yes	0.50	0.26 to 0.94	0.03	–	–	–	–	–	–

aOR, adjusted odds ratio; CI, confidence interval; TGW, transgender women; MSM, men who have sex with men; ART, antiretroviral therapy.

Models were run separately for overall, MSM and TGW. Factors showing significant level of 0.10 in univariate model were adjusted in multivariate models for each group. Factors not included in multivariate models were shown as – in this table.

Being TGW (OR 6.66, 95% CI 2.91 to 15.25, *p* < 0.001), spending 4 to 8 hours (OR 2.82, 95% CI 1.48 to 5.37, *p* = 0.002) or >8 hours (OR 2.33, 95% CI 1.05 to 5.16, *p* = 0.04) on social media per day, and having preference towards online services (OR 5.73, 95% CI 2.99 to 10.98, *p* < 0.001) and home‐based HIV testing (OR 6.00, 95% CI 3.1 to 11.63, *p* < 0.001) increased participant's likelihood to choose online HIV testing and post‐test counselling. However, having preference for immediate confirmatory HIV test and ART initiation (OR 0.32, 95% CI 0.15 to 0.67, *p* = 0.003), wanting an HIV testing technology to detect infection soonest post‐exposure (OR 0.30, 95% CI 0.16 to 0.57, *p* < 0.001), and having concerns around quality (OR 0.42, 95% CI 0.22 to 0.81, *p* = 0.01) or clinic hygiene (OR 0.31, 95% CI 0.16 to 0.6, *p* < 0.001) reduced selection chances.

### 3.4 Factors associated with selecting “Offline” and “Mixed” groups

Having a bachelor degree or higher (OR 0.5, 95% CI 0.25 to 0.98, *p* = 0.045), monthly income between 429 and 857 USD (OR 0.43, 95% CI 0.19 to 0.98, *p* = 0.04), and having tested for HIV twice or more (OR 0.39, 95% CI 0.16 to 0.97, *p* = 0.04) reduced the chance to select the Offline group. Being TGW (OR 0.03, 95% CI 0 to 0.26, *p* < 0.001) negatively influenced Mixed group selection, while having bachelor degree or higher (OR 3.10, 95% CI 1.48 to 6.46, *p* < 0.001), having had HIV tested twice or more (OR 5.90, 95% CI 2.09 to 16.64, *p* < 0.001), and high HIV prevention knowledge (OR 2.81, 95% CI 1.39 to 5.69, *p* < 0.001) positively influenced this selection.

### 3.5 Other factors associated with the decision to join the Online group and feelings post service‐utilization

Of the 208 participants who joined the Online group, 160 responded to the survey. For most (87.5%), it was an easy decision to choose online, supervised, HIV self‐testing. Preference for joining was guided by logistic/time convenience (46.9%), privacy and confidentiality (19.4%), altruism (16.9%), and scheduling flexibility (15.6%). Positive perceptions (“it's good and convenient”, “it's amazing”, “I'm glad to join HIV self‐testing”) increased from 67% before the process to 82% after. Negative perceptions (“fear of fingerprick”, “I feel anxious”, “I am nervous with the procedure”) decreased from 23.2% to 8.8%. Most agreed that the HIV self‐test kit mailed to them was complete (95%), the video and study brochure helped them comprehend the procedure (92.9%), and online video‐based guidance by counsellor while conducting self‐testing was satisfactory (95.1%).

### 3.6 Technology skills and utilization of counsellors and feasibility of providing online services

There were four MSM, one TGW and three cisgender women staff who responded to the survey. Median (IQR) age was 28.5 (24.5 to 31.5) years. Half had counselling experience of two years or less. Majority (75.0%) spent >4 hours using Internet in a day. All reported having used video calls for communication and felt comfortable using technology in their daily lives.

Smartphones (79.1%) and laptops (37.5%) were the primary devices used for delivering services. Primary apps for conducting video calls included LINE (87.5%), Facebook messenger (20.8%), ClickDesk (8.3%), Zoom (8.3%) and Facetime (8.3%). Majority (75%) reported experiencing a maximum of two Internet glitches per video call session. On a 5‐point Likert‐scale, majority (83.4%) rated the image quality of HIV self‐testing strip image as “very good” or “excellent” and all agreed that HIV test result image captured and displayed was sufficient and conclusive for providing post‐test counselling.

## 4 Discussion

Our implementation research study results illustrate that conducting online pre‐test counselling and online, supervised, HIV self‐testing and post‐test counselling among MSM and TGW are feasible in Thailand, when conducted by healthcare professionals and trained KP community health workers. Prior HIV testing experiences, privacy and confidentiality needs, HIV testing attitudes, and social network use patterns are significant factors driving the choice to select online, mixed or offline services. Online supervised HIV self‐testing significantly engaged first‐time testers and those with highest HIV prevalence, further emphasizing the need for large‐scale implementation of such model. Our study also demonstrated that an implementation research offering online, mixed and offline HIV testing options with self‐selection by participants is feasible and allows for near real‐life situations and lessons learned.

Having prior HIV testing experience may help facilitate one's decision to seek unconventional HIV testing services 18. MSM previously tested for HIV were more likely to choose online pre‐test counselling in our study. Inconvenient location and service hours are commonly cited barriers to scaling‐up HIV testing among MSM and other KP 8, 19. Those who cited such barriers were more likely to choose online pre‐test counselling, offering opportunities to foster participant‐counsellor relationship and enable appointment scheduling.

MSM participants with interest in home‐based testing and having concerns around confidentiality showed higher preference for online, supervised, HIV self‐testing, which supports findings among Chinese MSM who prioritized privacy and confidentiality when selecting online HIV services 20. In contrast to a previous study 21, seeking sex partners online did not influence participant's decision to seek online HIV testing. Although, consistent with an earlier finding, high social media usage increased participants’ selection of online HIV services 20.

Being TGW was another strong predictor for choosing online, supervised, HIV self‐testing in our study. High Internet and social media usage patterns among TGW and its potential to reach and provide non‐judgmental support to TGW was reported in earlier studies 22, 23, 24. TGW who considered taking an HIV test as a way to living healthy life were also more likely to choose online services. This is vital to designing public health interventions and research projects targeting TGW.

Our results harmonize well with previous study by Flowers et al. highlighting the need for diverse approaches to HIV testing interventions for maximum public health benefit 7. It is plausible that first‐time testers or testers with high risk would access a friendly offline HIV testing clinic where immediate confirmatory testing and linkages to PrEP/PEP or ART are available. Testers who perform regular check up every three to six months may prefer HIV self‐testing with an initial supervision by providers.

HIV self‐testing is not yet registered in Thailand as the Thai Food and Drug Administration lacks clarity around the level of support optimal for Thai users. Findings from this study and growing literature on the need for online counselling support for HIV self‐testers 10 should provide necessary guidance for shaping Thailand's national policy around online HIV self‐testing.

Concerns around quality of HIV services and intention to have immediate confirmatory testing and linkage to ART were main factors driving MSM and TGW away from selecting online HIV services. This may be particularly challenging for countries without current policies to regulate the quality, sale, distribution or use of HIV self‐test kits, commonly available online 25, 26. In addition, to implement online HIV services, efforts should be made to ensure that the KP communities clearly perceive high quality of online service delivery and trust that adequate support can be provided for linkage to clinical services.

Almost 40% of MSM and TGW in our study felt that the availability of NAT affected their decision to take HIV testing, pointing to the need for KPs’ access to an HIV testing assay which could allow for early detection of HIV infection. The availability of rapid 4th generation antigen‐antibody assay highlights the near‐future feasibility to have a self‐testing assay which could detect HIV infection earlier than the existing assays 27, 28.

Level of technology skills among healthcare providers has appeared to be the most important predictor of technology use in the workplace and is related to higher nursing competency 29, 30. Our findings showed that the level of technology ownership, skills and use was innately high among Thai providers who used them based on experience and self‐learning, and thus, did not require intensive technology use training to deliver online services. High satisfaction for online counselling guidance reported by participants, and satisfactory video and self‐testing result image quality expressed by providers have significant implications in terms of broader scale‐up of online, supervised, HIV self‐testing services in Thailand. However, among sub‐populations or in settings with inadequate digital literacy, efforts should focus on simplifying digital health technologies, which help to bridge, not exacerbate, health and social inequities 31.

Our study has a few limitations. Although participants were allowed to self‐select the groups, we could not fully avoid the possibility of bias by study staff at each study site who might have unintentionally influenced participants decision. All three options offered involve some level of social exposure and may not be inclusive enough for those who could not overcome even limited level of social exposure in the Online group. Secondly, due to unavailability of KPLHS clinics in other regions we did not enroll MSM and TGW beyond Bangkok Metropolitan Region and Pattaya to avoid possible bias to select the Online group. The result from this study, thus may not be generalized to MSM and TGW in other provinces. A recent online survey found that 30% of MSM in Southeast Asia had never been tested for HIV and were likely to be young, high‐risk, non‐gay‐identified MSM 32. Given these groups could be reached online for web‐based surveys, there is a high potential of such outreach platforms for delivering online HIV services. To reduce barriers in healthcare access, public health experts and programme implementers are encouraged to adapt online HIV service models explored in our study.

## 5 Conclusions

In summary, we demonstrated the feasibility of conducting online HIV counselling and testing services among Thai MSM and TGW. The online, supervised, HIV self‐testing service was particularly preferred by TGW, MSM who had privacy and confidentiality concerns, and those who spent more time using social media per day. Results from this study are vital in designing public health interventions targeting segments of MSM and TGW populations with preference towards online HIV services delivery.

## Competing interests

All authors declare no competing interest.

## Authors’ contributions

NP, TA and PP designed the study. NP led the study and wrote the first draft of the report. NP and TA edited and finalized the manuscript for submission. NP and DT designed the analysis. DT analysed the data. JJ, CN, KH and TS coordinated the study. PM oversaw data management. SP, PeP, ST, DM, SS, SH, and NU implemented the study at their sites. JB and TP supervised laboratory procedures. All authors critically reviewed and approved the manuscript.

## Funding

amfAR, The Foundation for AIDS Research as part of the GMT Initiative.
